# Disease burden of adverse childhood experiences across 14 states

**DOI:** 10.1371/journal.pone.0226134

**Published:** 2020-01-28

**Authors:** Geetha M. Waehrer, Ted R. Miller, Sara C. Silverio Marques, Debora L. Oh, Nadine Burke Harris

**Affiliations:** 1 Pacific Institute for Research and Evaluation, Calverton, Maryland, United States of America; 2 School of Public Health, Curtin University, Perth, Australia; 3 Center for Youth Wellness, San Francisco, California, United States of America; Stellenbosch University, SOUTH AFRICA

## Abstract

**Objective:**

To examine whether the relationship between Adverse Childhood Experiences (ACEs) and health outcomes is similar across states and persists net of ACEs associations with smoking, heavy drinking, and obesity.

**Methods:**

We use data from the Behavioral Risk Factor Surveillance System for 14 states. Logistic regressions yield estimates of the direct associations of ACEs exposure with health outcomes net of health risk factors, and indirect ACEs-health associations via health risk factors. Models were estimated for California (N = 22,475) and pooled data from 13 states (N = 110,076), and also separately by state.

**Results:**

Exposure to ACEs is associated with significantly higher odds of smoking, heavy drinking, and obesity. Net of these health risk factors, there was a significant and graded relationship in California and the pooled 13-state data between greater ACEs exposure and odds of depression, asthma, COPD, arthritis, and cardiovascular disease. Four or more ACEs were less consistently associated across states with cancer and diabetes and a dose-response relationship was also not present. There was a wide range across individual states in the percentage change in health outcomes predicted for exposure to 4+ ACEs. ACEs-related smoking, heavy drinking, and obesity explain a large and significant proportion of 4+ ACEs associations with COPD and cardiovascular disease, however some effect, absent of risk behavior, remained.

**Conclusions:**

ACE’s associations with most of the health conditions persist independent of behavioral pathways but only asthma, arthritis, COPD, cardiovascular disease, and depression consistently exhibit a dose-response relationship. Our results suggest that attention to child maltreatment and household dysfunction, mental health treatment, substance abuse prevention and promotion of physical activity and healthy weight outcomes might mitigate some adverse health consequences of ACEs. Differences across states in the pattern of ACEs-health associations may also indicate fruitful areas for prevention.

## Introduction

A growing body of research suggests that exposure to an accumulation of Adverse Childhood Experiences (ACEs) including abuse, neglect, parental substance abuse, or domestic violence creates an allostatic load that can lead to changes in neurological, immune, endocrine, and genetic regulatory systems, resulting in a negative physiological effect on health [1–7]. Studies show that severe, frequent, or prolonged adversity in childhood, without the buffering protection of a supportive caregiver, leads to changes in the structure and function of children’s developing brains resulting in an overactive stress response, impairment of executive functioning, difficulty with forming healthy relationships, and increased risky behaviors [8]. Much of the body’s stress response is mediated through hormones including adrenaline and cortisol, which exert their effects on virtually all of the body’s organ systems [9, 10]. Adverse childhood experiences are associated with chronic dysregulation of stress hormones as well as other hormones that regulate functions such as heart rate, blood pressure, metabolism, appetite, and reproduction [11–14].

In seminal research using data from a California health plan, ACEs were associated with an increased odds of adverse health outcomes including heart disease, liver disease, and lung disease, with a graded association between the number of ACEs and risk of some outcomes [15–19]. Subsequent studies have confirmed associations between exposure to ACEs and health outcomes in national data [20–23]. Additionally, population-level data demonstrate a dose-response relationship between ACEs exposure and adoption of health-damaging behaviors such as smoking, drinking, or disordered eating that also have negative health consequences [18, 23–25]. A recent meta-analysis reported that exposure to 4+ ACEs was weakly associated with physical inactivity, obesity and diabetes (odds ratios (OR) less than 2), moderately associated with smoking, heavy alcohol use, heart disease, respiratory disease, cancer (OR between 2 and 3), strongly associated with sexual risk-taking, poor mental health and problematic alcohol use (OR from 3 to 6), and had the strongest links with problem drug use and violence (OR more than 7) [26].

Gaps remain in our understanding of the relationship between ACEs and health. While several studies have found direct associations of ACEs with health outcomes net of risk behaviors like smoking (e.g., [17, 21]), the extent to which the ACEs-disease relationship runs through health risk factors is unclear. Although data from many jurisdictions have been analyzed, varying outcomes and demographic controls make it difficult to compare results. Results similar in direction and magnitude across multiple data sources would help build a case for causation. Given the differences in public health policy and environmental and socioeconomic differences across U.S. states, state differences in ACEs-health associations may also highlight fruitful areas for prevention.

The aims of this study are to estimate a uniform statistical model in 14 states (1) to assess the consistency of the association of ACEs with three health risk factors identified as leading causes of disease burden at the global level: smoking, heavy drinking, and obesity [27]; (2) to assess the consistency of the direct association of ACEs with a variety of health conditions, independent of associations with these health risk factors, and to analyze the indirect ACEs-health associations via these risk factors; and (3) to examine whether, net of health risk factors, the direct ACEs-health relationships differ across U.S. states. We examine these questions using data from U.S. Behavioral Risk Factor Surveillance System.

## Data

We used data from the Behavioral Risk Factor Surveillance System (BRFSS), the primary system of annual, cross-sectional, state-based random-digit-dialed telephone surveys of approximately 400,000 residents in the states and territories of the US. BRFSS uses a multistage sampling design to select a representative sample of the noninstitutionalized adult population aged 18 years and older residing within each state and territory. State health departments queried residents about demographics, risk behaviors, chronic health conditions, and use of preventive services using a standardized questionnaire developed in collaboration with the Centers for Disease Control (CDC). Each state survey includes the same core set of questions. Additional topical modules developed by the CDC are administered in some years by some states, probing topics such as arthritis, depression, and ACEs with state-developed questions of local interest also allowed. This study used BRFSS data from all 13 jurisdictions (12 states plus Washington, DC, henceforth referred to as the 13-state sample) that administered the CDC ACEs module between 2009 and 2012 and allowed CDC to post downloadable datasets. In addition, we obtained California Behavioral Risk Factor Survey data on ACEs for 2008, 2009, 2011, and 2013 from the Survey Research Group of the California Department of Public Health [28]. We analyzed data from California and CDC separately because the state has been unable to link the ACEs module data to CDC’s version of the CA BRFSS file (personal communication with Survey Research Group). In addition, the CDC’s changes to weighting (including raking weighting) were made at different times than CA complicating the task of analyzing the combination of the two datasets [29].

### Key variables

Health risk factors and health conditions were the main outcomes of interest in this study. We identified health risk factors using BRFSS self-reports of lifetime smoking, past-month heavy drinking (4 or more drinks for females or 5 or more drinks for males at one occasion), and obesity (body mass index greater than or equal to 30). For health conditions, we used self-reports of physician-diagnosed health conditions. For example, those with asthma were identified in BRFSS using the question, “Has a doctor, nurse, or other health professional ever told you that you had asthma”. We used similar questions to identify respondents with stroke, angina or coronary heart disease, chronic obstructive pulmonary disorder (COPD), arthritis, any cancer, diabetes, and depression. Respondents who reported a physician diagnosis of either a heart attack, stroke, or angina were identified as having “any cardiovascular disease”.

Exposure to ACEs was the main risk factor of interest in this study. The ACEs topical module (see online supplement S1 File) was adapted from the original CDC-Kaiser ACE study [18] and included 11 questions about childhood adversities experienced prior to age 18 [30] from which we created 8 indicators for ACEs as follows. As in previous studies [20, 23], we collapsed three frequency questions on sexual abuse (number of times forced to touch, be touched, or have sex with anyone at least 5 years older or an adult) into a single indicator for any sexual abuse, and two questions about frequency of physical and verbal abuse respectively into two indicator variables for any such abuse. We combined two questions on problem drinking and illegal drug use in the household into another indicator for household substance use. Four other indicators were created using affirmative responses to household dysfunction questions about parental divorce; living with a depressed or mentally ill person; living with a person who had been incarcerated; and frequency of physical violence between parents. The original ACEs studies indicated that childhood adversities did not occur in isolation. Instead, given exposure to one type of adversity, there was a very high likelihood of exposure to a second ACEs category [31, 32]. Thus, we focused on cumulative childhood stress as reflected in the number of ACEs and counted affirmative responses for the 8 components (parent divorce, incarceration, substance use, depression, domestic violence, child physical abuse, verbal abuse, sexual abuse) to construct an ACEs score ranging from 0 to 8 indicating the number of childhood adversities prior to age 18.

## Methods

We used the ACEs score to classify BRFSS respondents into four categories of ACE exposure (0 ACEs, 1 ACE, 2–3 ACEs, 4+ ACEs). Categorizing ACEs exposure in this way allows us to mitigate issues of sample size likely to arise for exposure to higher numbers of ACEs, especially in state-level analyses. We reported sample characteristics and the mean proportion of health outcomes for each ACEs category with 95% confidence intervals and used Wald tests to identify statistically significant differences between ACEs exposure categories.

### Logit models

We estimate logit models of health outcomes that control for ACEs exposure categories, with and without the health risk factors (lifetime smoking, heavy drinking, and obesity). Models including health risk factors will yield estimates of the direct association of ACEs with outcomes, net of risk factors. Models also control for race/ethnicity, sex, age, education, marital status, and employment. Education and employment are both imperfect proxies for childhood socioeconomic status and can control for differences in adult access to healthcare that can affect long-term health outcomes. State dummies were also included to control for regional differences in health access and behaviors that might be correlated with health outcomes. Finally, CDC changed their weighting methods in 2011 and redesigned the national BRFSS sample to include cellphone-only households. Similar changes were made to the California sample in 2012. Exploratory data analyses indicated that cellphone-only respondents have significantly more ACEs and are significantly less likely to have health insurance, be employed, or have completed high school than landline respondents, even after controlling for age. Lu and Slusky [33] reported that cellphone-only households also were at a greater distance from a health care provider. Thus, these respondents with ACEs may be more vulnerable to negative health outcomes in ways that are not fully captured by the control variables. To control for these differences, our regressions flag years when these weighting changes were in effect in our data (2011–12 for the 13-state sample and 2013 for CA) and include a dummy indicating which respondents only had cellphones.

### Indirect ACEs-health association via risk factors

To examine the extent to which the ACEs-health association runs through risk behaviors, prior studies have compared results from nested logistic regressions with sequential addition of risk factors [17]. As Karlson et al. [34] show, such a comparison of nested, same-sample, non-linear models does not yield correct estimates of the total (i.e. combined direct and indirect) ACEs-health association or the indirect association via risk factors because the coefficients of logit models are identified only up to scale and reflect both the underlying coefficient and the underlying residual standard deviation. Thus, when the health risk factors themselves are independently correlated with the health outcomes, coefficients of the nested models can differ both because of differences in the underlying parameter and because of differences in the residual standard deviation. We address this problem by expressing all the coefficients in the sparse logit models (i.e. without health risk factors) in the scale of the full model (i.e. including health risk factors). In this framework, a comparison of the ACEs coefficients with and without smoking, heavy drinking, and obese weight will indicate the extent to which ACEs association with health runs indirectly through these risk factors. Specifically, the indirect association of 4+ ACEs via these health risk factors is calculated as (ln(OR_sparse_)-ln(OR_full_))/ln(OR_sparse_) and presented as a percentage of the total 4+ACEs association with the different health outcomes. We estimate these logit models for CA and the pooled 13-state sample, and separately by state.

### Predicted change in probability

Since odds ratios are difficult to interpret and compare across different states, we also report the average predicted change in the probability of health outcomes for each category of ACEs exposure (1, 2–3, 4+) relative to 0 exposure. This is calculated as the difference in the predicted outcome probability for each individual in the sample with ACEs exposure first set to 0 and then set to each of the other three exposure categories, averaged over the whole sample. For each state, we calculate this predicted change in outcome probability due to 4+ ACEs as a percentage of the sample probability of the health outcome and report the median and the range of this relative change across all states.

### Dose-response relationship

For California and the pooled 13-state sample we use logit estimates from the full model including health risk factors to identify a dose-response relationship between ACEs exposure and health outcomes if (1) greater ACEs exposure is linked to a higher probability of the health outcome and (2) the increase in outcome probability for each exposure category relative to less exposure is statistically significant for at least 2 out of the 3 exposure categories.

### Final sample

Analyses were restricted to records with valid information on ACEs and complete information on socioeconomic characteristics. The samples included 110,076 respondents in the 13-state-sample and 22,475 respondents in CA respectively. Models for self-reported diagnosed asthma, depression, diabetes and the three health risk factors were estimated on those aged 18 and older; models for diseases with longer latency periods (COPD, arthritis, any cardiovascular disease, and cancer) were estimated on a sub-group of those aged 45 years and older. Sample sizes for individual health outcomes vary slightly because of missing or incomplete information. Modules for arthritis, COPD, depression, and cancer were only administered in a subset of states and years, also resulting in smaller analytical samples for these outcomes. Logit models were weighted and robust standard errors were calculated using the khb program in Stata 15 [35, 36].

## Results

### ACEs prevalence

Among 2009–12 BRFSS respondents in the 13-state sample, 38.3% reported no exposure to ACEs, 23.7% reported 1 ACE, 22.6% reported 2–3 ACEs, and 15.5% reported 4+ ACEs. In California, 39% reported 0 ACEs, 21.7% reported 1 ACE, 23.5% reported 2–3 ACEs, and 15.8% reported 4+ ACEs.

### Gender, race/ethnicity, age

Table 1 presents the characteristics of the 13-state BRFSS data by ACEs exposure. Compared to those with 0 ACEs, those reporting 4+ ACEs were significantly more likely to be female, less likely to be white, and more likely to be younger than 55 years old. There were significant differences in educational attainment by ACEs exposure. Those exposed to 4+ ACEs were significantly less likely to report a college degree (16.2%) compared to those with 0 ACEs (28.3%) and more likely to not complete high school (17.2% vs.10.5%). The unemployment rate among those with 0 ACEs (4.3%) was significantly lower than the rate for those exposed to any ACES, and a third of the unemployment rate for those exposed to 4+ACEs (13.2%).

**Table 1:** Sample characteristics by ACEs exposure (2009–2012 BRFSS).

	All	0 ACEs	1 ACE	2–3 ACEs	4 to 8 ACEs
**N**	110,076	46,210	25,604	23,627	14,635
**Sex**					
Male	43,860 (48.4%)	49.1%	50.9%*	48.9%	42.2%*
Female	66,216 (51.6%)	50.9%	49.1%**	51.1%	57.8%**
**Race/Ethnicity**					
White	89,678 (78.5%)	81.1%	78.7%**	76.3%**	75.2%**
Black	8,238 (9.5%)	7.9%	10.1%**	10.9%**	10.4%**
Hispanic	3,481 (5.1%)	4.0%	5.0%*	6.4%**	6.0%**
Other	8,679 (6.9%)	6.9%	6.2%	6.5%	8.4%**
**Age (years)**					
18–24	4,429 (11.6%)	9.3%	11.4%**	13.0%**	15.7%**
25–34	9,734 (16.9%)	13.3%	16.5%**	19.1%**	23.2%**
35–44	14,003 (18.0%)	14.7%	18.0%**	18.8%**	21.6%**
45–54	21,041 (19.5%)	18.4%	19.9%**	20.4%**	20.4%**
55–64	25,969 (16.3%)	17.8%	16.4%**	15.9%**	12.8%**
65+	34,900 (18.2%)	26.5%	17.8%**	12.8%**	6.4%**
**Marital Status**					
Married	60,197 (56.3%)	62.1%	56.6%**	52.9%**	46.5%**
Separated/Widowe d/Divorced	31,923 (18.5%)	18.5%	17.4%**	18.4%	20.3%**
Single	17,956 (25.2%)	19.3%	26.1%**	28.7%**	33.2%**
**Education**					
< High School	8,337 (10.9%)	9.4%	9.9%	11.3%**	15.3%**
HS/GED	31,821 (30.2%)	29.9%	29.9%	30.3%	31.2%
Some Post- Secondary	30,697 (31.4%)	29.5%	30.9%*	32.1%**	35.6%**
College Degree	39,221 (27.6%)	31.1%	29.2%**	26.3%**	18.0%**
**Employment**					
Employed	56,706 (58.3%)	55.9%	60.1%**	61.2%**	57.4%
Unemployed	5,910 (7.2%)	4.2%	6.9%**	8.7%**	13.0%**
Out of Labor Force	47,460 (34.5%)	39.9%	33.1%**	30.2%**	29.6%**

*p ≤ .05

**p ≤ .01

Table 2 shows the proportions of 13-state respondents in each ACEs category who report smoking, heavy drinking, obese weight, or health conditions diagnosed by a doctor or medical practitioner. Lifetime smoking, heavy drinking and obese weight were significantly more likely among those with some exposure to ACEs relative to those with 0 ACEs, as were depression, asthma, arthritis, and COPD. The proportion reporting these outcomes rose with greater exposure to ACEs. Compared to those with 0 ACEs, the prevalence of lifetime smoking and heavy drinking was 1.6 times as high among those with 4+ ACEs; the prevalence of obesity was 1.3 times as high; the prevalence of asthma was 2.1 times as high, arthritis was 1.3 times as high, COPD was 2.3 times as high, and the prevalence of depression was 4.1 times as high. For any cardiovascular disease, and diabetes, there were no significant differences in prevalence for those exposed to fewer than 4 ACEs. However, for those with 4+ ACEs, the prevalence of any cardiovascular disease was 1.12 times as high but the prevalence of diabetes was 0.88 times lower. The prevalence of cancer did not differ significantly by ACEs exposure.

**Table 2:** Prevalence of health risk factors and chronic disease by ACEs exposure.

Outcome	N^a^	%^b^	0 ACEs	1 ACE	2–3 ACEs	4+ACEs
**Health Risk Factors**						
**Lifetime Smoking**	109,628	46.30%	38.30%	43.80%**	51.70%**	61.90%**
		[45.7, 46.8]	[37.5, 39.1]	[42.7, 45.0]	[50.5, 52.8]	[60.4, 63.3]
**Heavy Drinking**	108,635	17.90%	14.10%	18.60%**	20.30%**	22.60%**
		[17.4, 18.3]	[13.5, 14.8]	[17.6, 19.5]	[19.4, 21.3]	[21.3, 24.0]
**Obesity**	105,514	28.80%	25.40%	29.60%**	30.90%**	33.10%**
		[28.4, 29.3]	[24.7, 26.1]	[28.6, 30.7]	[29.9, 32.0]	[31.7, 34.5]
**Chronic Disease**						
**Asthma**	109,630	12.30%	8.90%	11.80%**	13.80%**	19.10%**
		[11.9, 12.6]	[8.4, 9.4]	[11.0, 12.5]	[13.0, 14.6]	[18.0, 20.3]
**Arthritis,** **age≥45 yrs.**	66,619	40.00%	37.00%	40.20%**	42.50%**	47.50%**
		[39.4, 40.6]	[36.2, 37.9]	[38.9, 41.4]	[41.2, 43.9]	[45.6, 49.4]
**Any cardiovascular disease, age≥45 yrs.**	81,893	14.10%	13.80%	14.10%	14.10%	15.40%*
		[13.7, 14.5]	[13.2, 14.4]	[13.2, 14.9]	[13.2, 15.1]	[14.2, 16.7]
**Cancer,** **age≥45 yrs.**	67,011	18.00%	18.50%	17.70%	17.60%	17.30%
		[17.5, 18.4]	[17.9, 19.2]	[16.7, 18.6]	[16.5, 18.7]	[16.0, 18.7]
**COPD, age≥45 yrs.**	34,954	6.70%	4.80%	6.60%*	7.60%**	11.40%**
		[6.2, 7.2]	[4.2, 5.6]	[5.4, 8.1]	[6.6, 8.8]	[9.8, 13.3]
**Depression**	94,387	17.30%	8.90%	13.60%**	22.20%**	36.10%**
		[16.9, 17.7]	[8.4, 9.4]	[12.8, 14.4]	[21.2, 23.2]	[34.6, 37.6]
**Diabetes**	109,964	9.40%	9.80%	9.20%	9.40%	8.60%**
		[9.1, 9.6]	[9.4, 10.2]	[8.7, 9.8]	[8.9, 10.0]	[7.9, 9.3]

*p ≤ .05

**p ≤ .01

^a^ N is overall sample size in 2009–2012 BRFSS from 13 states, with available data on each outcome.

^b^ % is the prevalence of outcome.

### Adjusted odds of health outcomes

Table 3 reports on the adjusted odds ratios (OR) of health outcomes (with 95% confidence intervals) for different exposure to ACEs in the 13-state sample and in California. For each ACEs exposure category, Table 3 also reports the average predicted change in outcome probability relative to 0 ACES (in parentheses). Estimates for depression and chronic health conditions are shown both with and without adjustments for smoking, heavy drinking, and obesity. State-specific estimates of OR for health risk factors and health outcomes (independent of health risk factors), are presented in an online supplement S2 File. State-level results are also summarized in Table 4 which presents the number of BRFSS states with statistically significant OR for those exposed to 4+ ACEs, and the median and range across states in the percentage change in outcome probability predicted for exposure to 4+ ACEs relative to 0 ACEs.

**Table 3:** Association of ACEs exposure with health outcomes.

	Adjusted Odds Ratios (95% CI) and Predicted Change in Probability of Health Outcomes (Relative to 0 ACEs)								Dose-Response^b^	Indirect 4+ACEs Association as % of Total (*p* value)
			Total Association			Direct Association Net of Risk Factors				
	N	%	1 ACE	2–3 ACEs	4+ ACEs	1 ACE	2–3 ACEs	4+ ACEs		
**Health Risk Factors**										
**Lifetime Smoking**										
CA^a^	22,537	35.70%	1.49** (7.6) [1.32, 1.68]	1.83** (12.0) [1.62, 2.07]	2.86** (21.6) [2.47, 3.30]	-	-	-	Yes	
13 BRFSS States	109,628	46.30%	1.34** (6.4) [1.26, 1.42]	1.94** (14.8) [1.83, 2.06]	3.07** (24.8) [2.85, 3.30]	-	-	-	Yes	
**Heavy Drinking**										
CA	22,378	16.30%	1.28** (2.9) [1.07, 1.54]	1.50** (4.8) [1.25, 1.79]	1.95** (8.7) [1.60, 2.38]	-	-	-	Yes	
13 BRFSS States	108,635	17.90%	1.21** (2.4) [1.11, 1.32]	1.28** (3.2) [1.18, 1.40]	1.42** (4.6) [1.28, 1.57]	-	-	-	Yes	
**Obesity**										
CA	22,073	24.50%	1.06 (0.9) [0.93, 1.20]	1.17* (2.7) [1.03, 1.34]	1.48** (7.1) [1.28, 1.72]	-	-	-	Yes^c^	
13 BRFSS States	105,514	28.80%	1.23** (3.9) [1.15, 1.31]	1.32** (5.4) [1.24, 1.41]	1.45** (7.4) [1.34, 1.57]	-	-	-	Yes	
**Chronic Disease**										
**Asthma**										
CA	22,466	14.60%	1.17 (1.7) [0.99, 1.39]	1.36** (3.6) [1.16, 1.60]	1.96** (8.7) [1.64, 2.33]	1.15 (1.6) [0.97, 1.36]	1.32** (3.2) [1.13, 1.55]	1.82** (7.8) [1.53, 2.18]	Yes	10.4% (0.10)
13 BRFSS States	109,630	12.30%	1.32** (2.6) [1.20, 1.45]	1.52** (4.1) [1.39, 1.67]	2.05** (7.9) [1.85, 2.27]	1.28** (2.3) [1.16, 1.41]	1.44** (3.6) [1.31, 1.58]	1.87** (6.9) [1.69, 2.08]	Yes	12.2% (0.01)
**Arthritis, ≥45 yrs.**										
CA	12,448	35.20%	1.29** (4.9) [1.10, 1.50]	1.44** (7.2) [1.24, 1.67]	1.96** (13.7) [1.60, 2.40]	1.25** (4.4) [1.07, 1.46]	1.34** (5.7) [1.15, 1.55]	1.74** (11.3) [1.43, 2.13]	Yes	17.5% (0.12)
10 BRFSS States	66,619	40.00%	1.30** (5.4) [1.21, 1.39]	1.52** (8.9) [1.42, 1.64]	1.97** (14.5) [1.80, 2.16]	1.25** (4.7) [1.17, 1.34]	1.43** (7.5) [1.33, 1.53]	1.80** (12.6) [1.64, 1.97]	Yes	13.5% (0.03)
**Depression**										
CA	17,722	13.40%	1.89** (4.9) [1.51, 2.36]	3.01** (10.1) [2.49, 3.64]	5.50** (19.4) [4.48, 6.76]	1.83** (4.7) [1.47, 2.29]	2.88** (9.8) [2.37, 3.49]	4.98** (18.0) [4.03, 6.15]	Yes	5.8% (0.06)
10 BRFSS States	94,387	17.30%	1.61** (4.6) [1.47, 1.77]	2.93** (12.7) [2.69, 3.18]	5.38** (23.6) [4.91, 5.90]	1.54** (4.3) [1.41, 1.69]	2.67** (11.6) [2.45, 2.91]	4.65** (21.4) [4.24, 5.11]	Yes	8.6% (0.00)
**COPD, ≥45 yrs.**										
CA	9,096	6.60%	1.78** (2.6) [1.28, 2.49]	2.70** (5.3) [2.01, 3.62]	3.13** (6.5) [2.18, 4.48]	1.63** (2.3) [1.17, 2.28]	2.33** (4.6) [1.73, 3.14]	2.42** (4.8) [1.68, 3.48]	Yes	22.5% (0.02)
5 BRFSS States	34,954	6.70%	1.48** (1.9) [1.16, 1.88]	1.84** (3.2) [1.46, 2.31]	2.86** (6.6) [2.21, 3.69]	1.33* (1.5) [1.05, 1.70]	1.47** (2.1) [1.17, 1.85]	2.00** (4.2) [1.55, 2.59]	Yes	33.84% (0.01)
**Any Cardiovascular Disease, ≥45 yrs.**										
CA	15,963	11.50%	1.25* (1.9) [1.03, 1.51]	1.44** (3.3) [1.19, 1.75]	1.74** (5.3) [1.35, 2.24]	1.20 (1.6) [0.99, 1.45]	1.36** (2.8) [1.12, 1.65]	1.56** (4.2) [1.21, 2.01]	Yes^d^	19.83% (0.08)
13 BRFSS States	81,893	14.10%	1.16** (1.5) [1.06, 1.26]	1.27** (2.5) [1.16, 1.40]	1.64** (5.7) [1.45, 1.84]	1.12* (1.2) [1.03, 1.22]	1.19** (1.9) [1.08, 1.31]	1.47** (4.4) [1.31, 1.66]	Yes	21.21% (0.01)
**Diabetes**										
CA	22,450	9.00%	1.12 (0.8) [0.92, 1.36]	0.93 (-0.5) [0.78, 1.11]	1.27* (1.8) [1.03, 1.56]	1.10 (0.7) [0.91, 1.34]	0.90 (-0.7) [0.75, 1.08]	1.17 (1.2) [0.95, 1.45]	No	32.75% (0.36)
13 BRFSS States	109,964	9.40%	1.09 (0.6) [0.99, 1.18]	1.24** (1.6) [1.14, 1.36]	1.30** (2.0) [1.17, 1.46]	1.05 (0.3) [0.96, 1.14]	1.18** (1.2) [1.08, 1.29]	1.21** (1.4) [1.09, 1.35]	Yes^d^	27.71% (0.38)
**Cancer, ≥45 yrs.**										
CA	6,817	16.40%	1.22 (2.5) [0.98, 1.53]	1.16 (1.8) [0.92, 1.46]	1.15 (1.8) [0.85, 1.56]	1.22 (2.5) [0.98, 1.52]	1.14 (1.7) [0.90, 1.45]	1.13 (1.5) [0.83, 1.54]	No	16.35% (0.63)
10 BRFSS States	67,011	18.00%	1.09* (1.2) [1.01, 1.18]	1.20** (2.6) [1.10, 1.32]	1.35** (4.2) [1.20, 1.51]	1.09* (1.1) [1.00, 1.17]	1.19** (2.4) [1.09, 1.30]	1.31** (3.8) [1.17, 1.47]	Yes	8.3% (0.06)

*p ≤ .05

**p ≤ .01

Baseline models control for age, sex, race/ethnicity, education, employment status, marital status, state dummies, two dummies respectively for cellphone-only respondents and for the year when sampling changes were in effect in the BRFSS (2011–12 in the 13-state sample and 2013 in CA data), and flags for missing information.

^a^ California (CA) data come from 2008, 2009, 2011, and 2013.

^b^ Dose-response identified if (1) greater ACEs exposure is linked to higher probability of outcome and (2) the increase in probability of outcome is statistically significant (p<0.10) for at least 2 out of the 3 comparisons: 1 ACE vs. 0 ACE; 2–3 ACEs vs. 1 ACE; 4+ ACEs vs 2–3 ACEs.

^c^ For obesity, dose response identified by statistically significant increase in outcome probability for 2–3 ACEs vs 0 ACEs and 4+ ACEs vs 2–3 ACEs. For cardiovascular disease, dose-response identified by significant increases between 2–3 ACEs vs 1 ACE and 4+ ACEs vs 1 ACE. For diabetes, significant increases between 2–3 ACEs vs 1 ACE and 4+ ACEs vs 1 ACE.

**Table 4:** Summary of results across 14 states.

	Number of States Analyzed	With Statistically Significant^a^ OR for those with 4+ ACEs	Median % Change in Outcome Probability for 4+ ACEs	Range Across States in % Change in Outcome Probability for 4+ ACEs
**Health Risk Factors**				
**Lifetime Smoking**	14	14	52%	37%-67%
**Heavy Drinking**	14	9	26%	2%-53%
**Obesity**	14	12	28%	10%-56%
**Chronic Disease**				
**Asthma**	14	14	56%	35%-87%
**Arthritis, ≥45 yrs.**	11	10	32%	15%-45%
**Any Cardiovascular Disease, ≥45 yrs.**	14	10	33%	15%-63%
**Cancer, ≥45 yrs.**	11	3	13%	-12%-32%
**COPD, ≥45 yrs.**	6	5	76%	44%-116%
**Depression**	11	11	124%	110%-165%
**Diabetes**	14	4	16%	-32%-44%

Data includes California and Washington, DC. Full set of state-level odds ratios (OR) and 95% CI for states in the 13-state sample available in Online Supplement S2 File.

^a^ *p* ≤ .05

### Health risk factors

Lifetime Smoking: Compared to those with 0 ACEs, the adjusted odds of lifetime smoking were 186% higher in California and 207% times higher for those with 4+ ACEs in the 13-state sample. In both data, the average predicted change in smoking probability increases with ACEs exposure. For example, the change in the average predicted probability of lifetime smoking in California increases from 7.6 percentage points for exposure to 1 vs. 0 ACEs to 12 percentage points for exposure to 2–3 ACEs vs. 0 ACEs, and 21.6 percentage points for exposure to 4+ ACEs vs 0 ACEs. This increase is statistically significant for at least two of three ACEs categories suggesting a dose-response relationship. At the state level, the odds of smoking for those with 4+ ACEs was significantly greater across all 13 states (Table 4 and online supplement File for state-level ORs).

Heavy Drinking: Relative to those with 0 ACEs, the adjusted odds of heavy drinking were 95% higher for those reporting 4+ ACEs in California and 42% higher in the 13-state sample with a dose-response relationship in both data. However, state-level estimates show an inconsistent picture with no significant associations between 4+ ACEs and heavy drinking in five BRFSS states (Arkansas, Washington DC, Iowa, Nevada, Louisiana).

Obesity: Compared to those reporting zero ACEs, the adjusted odds of obese weight for those reporting 4+ ACEs was approximately 45–50% higher in both California and the pooled 13-state sample with a dose-response relationship. The adjusted odds of obesity was significantly higher for those reporting 4+ ACEs exposure in 11 of the 13 states in the sample.

### Health conditions

Results presented below focus on the direct associations of ACEs with health conditions independent of lifetime smoking, heavy drinking and obese weight.

Asthma: Relative to those with 0 ACEs, 4+ ACEs was directly associated with 82% higher odds of reporting asthma in California (CA) and had 87% higher odds in the 13-state sample, with a dose-response relationship in both samples. Results by state showed similar patterns.

Arthritis: Relative to those 45 years and older reporting 0 ACEs, those with 4+ ACEs had 74% higher odds of an arthritis diagnosis in CA and 80% higher odds in 10 BRFSS states with arthritis data, with a dose-response relationship in both samples. Results by state showed significantly higher arthritis odds for 4+ ACEs in all states except one (Tennessee).

Depression: Relative to those with 0 ACEs, those reporting 4+ ACEs had almost 400% higher odds of depression in CA and 365% higher odds in the 10 BRFSS states with depression data, with a dose-response relationship in both data. Results by state confirmed these patterns.

COPD: Relative to those 45 years and older with 0 ACEs, those reporting 4+ ACEs have a 142% higher odds of COPD in California and 100% higher odds in the 5 states with BRFSS data with a dose-response relationship. Results at the state level show a similar pattern. However, including asthma in the models reduces odds ratios and weakens evidence of a dose-response relationship (results available upon request).

Any Cardiovascular Disease: Relative to those 45 years and older with 0 ACEs, those reporting 4+ ACEs had 56% higher odds of any cardiovascular disease in CA and 47% higher odds in the 13-state sample, with a dose-response relationship. However, state-level analyses showed no statistically significant 4+ ACEs association with any cardiovascular disease in 4 states (Arkansas, Louisiana, Nevada, Tennessee).

Cancer: California data did not show significant differences in the odds of cancer by ACEs exposure. However, in the 10 BRFSS states with cancer data, those aged 45 years and older who reported 4+ ACEs had 31% higher odds of cancer relative to 0 ACEs with a dose-response relationship. Relative to those reporting 0 ACEs, state-level analyses show wide variation in the odds of cancer for those with 4+ ACEs, ranging from 15% lower odds (Arkansas) to 50% higher odds (Vermont). Only 3 states (Vermont, Washington, Wisconsin) had significantly higher odds of cancer for those with 4+ ACEs.

Diabetes: California data showed no significant association between ACEs exposure and odds of diabetes. Those reporting 4+ ACES had 21% higher odds of diabetes than those with 0 ACEs in the 13-state sample, with a modest dose-response relationship. State-level estimates showed that 4+ ACEs were significantly associated with diabetes odds in only four states with odds ratios ranging from 36% lower diabetes odds (Hawaii) to 75% higher odds (Iowa) for those reporting 4+ ACEs.

### Indirect association via risk factors

A comparison of the total and direct associations of 4+ ACEs with odds of health conditions indicate significant indirect ACEs associations via smoking, heavy drinking and obese weight. For example, in the 13-state sample, these health risk factors account for approximately 12% of the total 4+ ACEs association with asthma, 14% of the association with arthritis, 9% of the association with depression, approximately 21% of the total association any cardiovascular disease, and 34% of the total 4+ACEs association with COPD.

### Relative change in outcome probability associated with 4+ ACEs

Table 3 shows that a change in ACEs from no exposure to 4+ ACEs is associated with 21.6 percentage point increase in the average predicted probability of lifetime smoking in California, equivalent to a 60% change relative to the sample smoking probability (calculated as 21.6 divided by 36). Similarly, we estimate relative change in outcome probability associated with 4+ ACEs for the other BRFSS states and report the median and range of relative change across the 14 states (including California) in Table 4. Table 4 also presents the number of states in our data with statistically significant OR for those exposed to 4+ ACEs.

Among health risk factors, a change from 0 to 4+ ACEs is associated with 52% median estimate of the relative change in smoking probability, much higher than the median estimates for obesity (28%) and heavy drinking (26%). Among chronic health conditions, exposure to 4+ ACEs is associated with the largest median relative change for depression (124%), followed by COPD (76%), asthma (56%), any cardiovascular disease (33%), arthritis (32%), diabetes (16%), and any cancer (13%). Table 4 also shows a wide range across states in estimates of the relative change in outcome probabilities. Thus, the relative change in heavy drinking probability is predicted to range from an insignificant 2% (Arkansas) to 53% (California) for an increase in ACEs exposure from 0 to 4+ ACEs and the relative change in asthma probability ranges from 35% (North Carolina) to 87% (Washington, DC). Exposure to 4+ ACEs was predicted to at least double the probability of depression in all the states with data, associations with the probability of COPD ranged from 44% (Wisconsin) to 116% (Vermont). In contrast, the probabilities of diabetes and cancer were predicted to fall with exposure to 4+ ACEs in some states. Among health risk factors, the ratio of the highest and lowest state estimates of relative change in probability is the largest for heavy drinking (26.2), followed by obesity (5.6), and lifetime smoking (1.8). Among health outcomes, the high/low ratio is the largest for cardiovascular disease (4.2), followed by arthritis (3), COPD (2.6), asthma (2.5), and depression (1.5) (excluding diabetes and cancer where exposure to 4+ ACEs is predicted to lower probabilities).

## Discussion

Studies show that early life stress is associated with long-term changes to the immune system leading to increased measures of inflammation and impairments of certain immune functions [37, 38]. In addition, childhood adversity is associated with changes to genetic regulatory mechanisms leading to long-term changes in the way the brain and immune system respond to stress as well as premature cellular aging with physiological effects on bodily functions including blood pressure, heart rate, and metabolism [11–14]. Recent animal studies show that chronic stress can alter the balancing of costs and benefits and result in impulsive actions and risky behaviors [39, 40]. This growing body of research indicates the biological pathways by which exposure to ACEs can affect health outcomes, both directly and indirectly via health risk behaviors.

Our analysis of data from 13 states and California data finds that those exposed to ACEs have higher odds of obesity, smoking, heavy drinking, depression, asthma, arthritis, COPD, and cardiovascular disease with a dose-response relationship between the reported number of ACEs and these outcomes. State-level analyses also showed significant associations between ACEs exposure and the odds of these conditions in most of the states examined. There was no significant association between ACEs exposure and cancer or diabetes in California but those exposed to ACEs had significantly higher odds of these diseases with a dose-response relationship in the pooled 13-state sample. At the same time, separate analyses of these outcomes in each of the 13 states yielded significant associations of cancer and diabetes with 4+ ACEs in only 3 or 4 states, and associations were even negative in some states. This pattern of results suggests that beyond links through risk factors, the relationship between ACEs exposure and cancer or diabetes is at best modest.

Our 13-state pooled data yield baseline ORs for asthma, and any cardiovascular disease that are generally consistent with Gilbert et al. [23] who used 2010 BRFSS data albeit with a slightly different ACEs categorization. Total associations of smoking, obesity, and COPD (respiratory disease) with 4+ ACEs are generally consistent with results from a meta-analysis by Hughes et al. while our mixed findings for diabetes are consistent with the weak results in that study. Bellis et al. [41] also found insignificant associations between ACEs exposure and diagnoses of cancer or diabetes. Moreover, the relative odds of cardiovascular disease, COPD, diabetes, and cancer for those with 4+ ACEs are smaller in our study than in the seminal ACEs studies which focused on a sample of mostly middle-class white adults in California. For example, Felitti et al. [18], which did not control for health risk factors, estimated that for those reporting 4+ ACEs the odds of diabetes were 1.6 times higher, COPD odds were 3.9 times higher, and cancer odds were 1.9 times higher compared to those reporting 0 ACEs. By comparison, our California analyses indicated odds ratios of 1.3 for diabetes, 3.1 for COPD, and no significant ACEs association with cancer, and odds ratios were even smaller when models adjusted for ACEs associations with health risk factors. These differences and the lack of significant ACEs associations with cancer persisted even when the California models were re-estimated using all adults (results available upon request).

Our results from California and the 13-state sample show that associations of 4+ ACEs with most of the health conditions persist independent of behavioral pathways but only asthma, arthritis, COPD, cardiovascular disease, and depression consistently exhibit a dose-response relationship that could conceivably strengthen the case for a causal interpretation of the results. ACEs-related smoking, heavy drinking and obesity explain a significant proportion of the previously observed elevations in chronic disease odds associated with ACEs exposure. This pathway is especially strong for any cardiovascular disease, and COPD. Sensitivity analyses indicate that a combination of health risk factors and asthma explain 46% of the increased COPD odds associated with 4+ ACEs exposure. Our results suggest smoking, alcohol misuse, and obesity are important pathways by which ACEs may affect lifelong health, offering important opportunities for preventive strategies to mitigate the health consequences of ACEs.

We found significant state variation in ACEs associations with health outcomes with a wide range across the 14 states (including California) in the relative change in outcome probability predicted to follow a change in ACEs exposure from 0 to 4+ ACEs. Such differences may indicate that unobserved regional characteristics are correlated with both ACEs exposure and health outcomes. For example, Washington, DC has the highest change in asthma probability (87%) predicted in response to exposure to 4+ ACEs which may reflect higher pollution in this urban jurisdiction that may exacerbate the influence of ACEs on respiratory health. Similarly, greater access to health care or higher taxes on alcohol or cigarettes could also moderate ACEs impact on health behaviors and outcomes. While an exploration of the reasons for state differences in ACEs-health associations is beyond the scope of our study, future longitudinal studies could differentiate states by these and other state policies on child welfare or parental leave/bonding and examine whether such policy levers may moderate the impact of ACEs on longer-term health outcomes.

Prevention of ACEs-attributable illness requires screening for ACEs and collaboration among medical, behavioral health, and public health experts treating their effects. Primary care professionals can integrate universal screening for ACEs in their clinics, refer patients to mental health or multi-disciplinary treatment, and closely monitor and treat the development of behaviors and illnesses associated with ACEs. Screening for ACEs in childhood provides ways to reduce exposure and intervene early, while screening in adult primary care can inform and shape disease prevention and management activities. Screening of childhood adversities in primary care has proven effective in increasing quality of care while reducing healthcare costs and maltreatment prevalence [39–46]. Mental health treatment also has been effective [47, 48]. Clinical integration of screening for early life adversity is important [49–51] In addition to screening, health care providers should address behavioral risks associated with ACEs, including alcohol and drug use, smoking, weight problems, and eating disorders [17, 19, 24, 52–58].

This study has several limitations. First, the retrospective ACEs data are self-reported and subject to recall bias. Studies show only modest concordance between retrospective and prospective data on ACEs [42, 59], with retrospective ACEs data more predictive of health outcomes measured subjectively (i.e., self-reported as in BRFSS) while prospective ACEs data collected in childhood are more predictive of health outcomes measured objectively with tests and biomarkers [60]. Studies on child sexual abuse show that reporting varies according to the definition of abuse and mode of survey administration, with rates reported by children living at home lower than their retrospective reports as adults [61]. To the degree that child abuse is under- or over-reported, our estimates may misstate ACEs associations with outcomes. Second, the standard ACEs module in the BRFSS lacks questions on physical or emotional neglect that were part of the ACEs count in the second wave of the original CDC-Kaiser study [17]. That exclusion may result in lower cumulative ACE scores and cause us to underestimate ACEs associations with health outcomes. Third, while our results highlight ACEs associations with health behaviors and outcomes, the timing and duration/intensity of ACEs is not indicated in the cross-sectional BRFSS data especially in relation to onset of asthma, smoking, or heavy drinking, limiting a causal interpretation of our results. Fourth, while our study estimates ACEs associations with health risk behaviors, we did not examine all the health outcomes resulting from risky behaviors, such as HIV status, due to lack of data. Fifth, the data do not include information about childhood background including parental socioeconomic status or parental health conditions, nor is there information on other traumatic life events. To the extent that these are correlated with both the number of ACEs and chronic health conditions, our measures of ACEs associations with health outcomes may be biased estimates of any causal impact on long-term health. Information on childhood residence was also lacking, precluding analysis of any protective effects of state policies that mitigate the negative impacts of ACEs on the adoption of health risk behaviors. Finally, similar to other ACEs studies using BRFSS data, this study relies on self-reports of doctor-diagnosed health conditions and self-reports of health risk behaviors. Studies have found that survey respondents underestimate their weight, under-report alcohol and tobacco use, and chronic conditions may go undiagnosed for a long time [62–65]. Bowlin et al. [52] found that the prevalence of obesity and smoking in BRFSS was underreported. However, this and other studies have also found moderate sensitivity in BRFSS self-reports of diabetes and arthritis [53, 54] and BRFSS heart disease prevalence has been treated as a benchmark in another study [55]. Still, to the extent that self-reports underestimate the prevalence of the health conditions and risk factors, we may underestimate their associations with ACEs.

Despite these limitations, this study takes a step closer to understanding the relationship between ACEs and health outcomes, net of ACEs associations with smoking, heavy drinking, and obesity. We find a direct association with asthma, arthritis, COPD, cardiovascular disease, and depression across states, with a clear dose-response relationship. However, the evidence for such a direct ACEs association is less convincing for cancer and diabetes. Prospective data would greatly improve the task of establishing a causal relationship between Adverse Childhood Experiences and negative health outcomes. More work is needed to understand state differences in ACEs associations with health and the social, health, and economic supports that can mitigate the effect of childhood adversities on later-life outcomes.

## Supporting information

S1 File BRFSS Adverse Childhood Experience (ACE) Module. (PDF)

S2 File State estimates of direct association of ACEs exposure with health outcomes (adjusted odds ratios using BRFSS data in 13 states). (PDF)
