# Supplementary material for: Disease burden of adverse childhood experiences across 14 states
Source: PLoS One. 2020 Jan 28;15(1):e0226134. doi: 10.1371/journal.pone.0226134 (PMC6986706; doi:10.1371/journal.pone.0226134)
Supplement: S1 File — (PDF) [file pone.0226134.s001.pdf]

BRFSS Adverse Childhood Experience (ACE) Module

Prologue: I'd like to ask you some questions about events that happened during your childhood. This information will allow us to better understand problems that may occur early in life, and may help others in the future. This is a sensitive topic and some people may feel uncomfortable with these questions. At the end of this section, I will give you a phone number for an organization that can provide information and referral for these issues. Please keep in mind that you can ask me to skip any question you do not want to answer. All questions refer to the time period before you were 18 years of age. Now, looking back before you were 18 years of age---.

1) Did you live with anyone who was depressed, mentally ill, or suicidal?

2) Did you live with anyone who was a problem drinker or alcoholic?

3) Did you live with anyone who used illegal street drugs or who abused prescription medications?

4) Did you live with anyone who served time or was sentenced to serve time in a prison, jail, or other correctional facility?

5) Were your parents separated or divorced?

6) How often did your parents or adults in your home ever slap, hit, kick, punch or beat each other up?

7) Before age 18, how often did a parent or adult in your home ever hit, beat, kick, or physically hurt you in any way? Do not include spanking. Would you say—

8) How often did a parent or adult in your home ever swear at you, insult you, or put you down?

9) How often did anyone at least 5 years older than you or an adult, ever touch you sexually?

10) How often did anyone at least 5 years older than you or an adult, try to make you touch sexually?

11) How often did anyone at least 5 years older than you or an adult, force you to have sex?

Response Options

<u>Questions 1-4</u>
1=Yes
2=No
7=DK/NS
9=Refused

<u>Question 5</u>
1=Yes
2=No
8=Parents not married
7=DK/NS
9=Refused

<u>Questions 6-11</u>
1=Never
2=Once
3=More than once
7=DK/NS
9=Refused

CDC. 2018. BRFSS Adverse Childhood Experience (ACE) Module. Available at https://www.cdc.gov/violenceprevention/acestudy/pdf/BRFSS_Adverse_Module.pdf
