# Supplementary material for: Disease burden of adverse childhood experiences across 14 states
Source: PLoS One. 2020 Jan 28;15(1):e0226134. doi: 10.1371/journal.pone.0226134 (PMC6986706; doi:10.1371/journal.pone.0226134)
Supplement: S2 File — (PDF) [file pone.0226134.s002.pdf]

Table S2. Online Supplement: State Estimates of Direct Association of ACEs Exposure with Health Outcomes (Adjusted Odds Ratios using BRFSS data in 13 states)

State	Years	N[a]	%	1 ACE	2-3 ACE	4+ ACE	N	%	1 ACE	2-3 ACE	4+ ACE	N	%	1 ACE	2-3 ACE	4+ ACE
Lifetime Smoking							Heavy Drinking					Obese				
Arkansas	2009	3,586	49.20%	1.53** [1.17, 1.99]	1.56** [1.18, 2.05]	2.57** [1.76, 3.76]	3,552	10.90%	1.67* [1.03, 2.70]	1.36 [0.81, 2.30]	1.03 [0.54, 1.95]	3,442	31.90%	1.31 [0.99, 1.74]	1.20 [0.90, 1.60]	1.30 [0.90, 1.88]
Louisiana	2009	8,201	45.80%	1.23* [1.03, 1.45]	1.80** [1.50, 2.17]	2.89** [2.29, 3.64]	8,112	14.60%	0.91 [0.69, 1.21]	1.02 [0.77, 1.35]	1.03 [0.73, 1.45]	7,938	33.90%	1.20* [1.01, 1.42]	1.27* [1.04, 1.51]	1.36** [1.07, 1.71]
N.Carolina	2012	10,696	46.50%	1.20** [1.04, 1.38]	1.81** [1.57, 2.10]	2.74** [2.30, 3.26]	10,420	12.60%	1.35* [1.06, 1.72]	1.52** [1.19,	1.70** [1.31, 2.20]	10,141	30.10%	1.18* [1.02, 1.37]	1.39** [1.19, 1.62]	1.60** [1.34, 1.91]
Tennessee	2012	5,618	49.10%	1.41** [1.14, 1.75]	2.01** [1.63, 2.47]	2.77** [2.15, 3.56]	5,525	10.80%	1.48 [0.98, 2.21]	1.31 [0.89, 1.94]	1.74** [1.15, 2.63]	5,373	32.40%	1.08 [0.87, 1.33]	1.04 [0.84, 1.29]	1.16 [0.89, 1.50]
Washington DC	2010	3,513	38.80%	1.43** [1.12, 1.82]	1.51** [1.18, 1.93]	1.94** [1.41, 2.66]	3,479	16.50%	1.09 [0.76, 1.56]	1.32 [0.94, 1.85]	1.43 [0.94, 2.19]	3,436	21.60%	1.13 [0.81, 1.57]	1.13 [0.83, 1.53]	1.71** [1.17, 2.52]
Vermont	2010-11	12,907	47.70%	1.26** [1.12, 1.43]	1.59** [1.40, 1.81]	2.70** [2.30, 3.16]	12,845	17.90%	1.03 [0.85, 1.27]	1.13 [0.93, 1.38]	1.25* [1.00, 1.56]	12,512	25.00%	1.12 [0.97, 1.29]	1.24** [1.08, 1.44]	1.51** [1.28, 1.79]
Iowa	2012	6,214	42.70%	1.41** [1.19, 1.67]	2.09** [1.75, 2.49]	3.56** [2.86, 4.43]	6,176	21.40%	1.23 [0.98, 1.55]	1.2 [0.95, 1.52]	1.05 [0.79, 1.39]	5,945	30.70%	1.1 [0.92, 1.32]	1.16 [0.96, 1.39]	1.36** [1.09, 1.70]
Wisconsin	2010-12	13,133	46.80%	1.35** [1.17, 1.55]	2.20** [1.90, 2.55]	3.27** [2.72, 3.93]	13,048	23.60%	1.16 [0.97, 1.39]	1.24* [1.03, 1.49]	1.28* [1.02, 1.61]	12,607	28.50%	1.32** [1.14, 1.54]	1.33** [1.14, 1.56]	1.42** [1.16, 1.73]
Minnesota	2011	13,445	45.60%	1.41** [1.24, 1.61]	2.01** [1.74, 2.31]	4.03** [3.38, 4.82]	13,331	22.30%	1.19 [0.99, 1.43]	1.09 [0.90, 1.31]	1.42** [1.15, 1.76]	12,827	26.00%	1.22* * [1.05,	1.43** [1.22, 1.68]	1.59** [1.32, 1.92]
Montana	2011	9,146	48.50%	1.12 [0.78, 1.60]	1.46* [1.04, 2.04]	3.08** [2.14, 4.44]	9,088	21.10%	1.01 [0.80, 1.28]	1.65** [1.32,	1.43** [1.10, 1.86]	8,886	24.90%	1.16 [0.95, 1.42]	1.08 [0.88, 1.32]	1.33* [1.06, 1.66]
Nevada	2010	3,490	47.70%	1.12 [0.78, 1.60]	1.46* [1.04, 2.04]	3.08** [2.14, 4.44]	3,440	17.40%	1.07 [0.65, 1.75]	1.26 [0.78, 2.04]	1.58 [0.98, 2.55]	3,396	22.60%	1.17 [0.82, 1.67]	1.99** [1.39, 2.84]	1.96** [1.31, 2.93]
Hawaii	2010	6,117	39.70%	1.52** [1.24, 1.86]	1.84** [1.49, 2.28]	3.32** [2.55, 4.32]	6,081	17.90%	1.50** [1.11,	1.39* [1.02, 1.90]	1.85** [1.31, 2.61]	6,050	22.70%	1.27 [1.00, 1.63]	1.55** [1.21, 1.98]	2.06** [1.52, 2.78]
Washington	2011	13,562	44.30%	1.36** [1.17, 1.59]	2.06** [1.76, 2.41]	3.00** [2.50, 3.60]	13,538	17.30%	1.34* [1.06, 1.70]	1.61** [1.29,	1.99** [1.56, 2.54]	12,961	27.10%	1.32** [1.12, 1.57]	1.43** [1.21, 1.69]	1.47** [1.21, 1.77]
		Asthma					Arthritis					Depression				
Arkansas	2009	3,574	11%	1.34 [0.88, 2.02]	1.62* [1.01, 2.60]	1.79* [1.08, 2.97]	2,823	45.30%	1.28* [1.01, 1.63]	1.73** [1.32,	1.79** [1.25, 2.58]	-				
Louisiana	2009	8,185	10.70%	1.30 [0.98, 1.73]	1.44* [1.08, 1.93]	1.75** [1.26, 2.44]	6,140	40.60%	1.14 [0.96, 1.35]	1.51** [1.24,	1.85** [1.42, 2.39]	-				
N.Carolina	2012	10,732	11.60%	1.28* [1.02, 1.61]	1.29* [1.02, 1.64]	1.49** [1.16, 1.92]	7,486	41.60%	1.22* [1.04, 1.44]	1.39** [1.16,	2.00** [1.60, 2.49]	10,720	17.80%	1.38** [1.13, 1.69]	2.18** [1.80, 2.64]	4.61** [3.75, 5.67]
Tennessee	2012	5,639	10.70%	1.47* [1.00, 2.17]	1.47* [1.02, 2.12]	1.53* [1.04, 2.25]	4,217	44%	0.96 [0.76, 1.21]	1.24 [0.98, 1.57]	1.34 [1.00, 1.81]	5,633	20.00%	1.29 [0.98, 1.69]	2.96** [2.30, 3.80]	4.45** [3.38, 5.86]
Washington DC	2010	3,503	16%	1.45* [1.01, 2.10]	1.80** [1.30, 2.51]	2.80** [1.89, 4.14]	-					-				
Vermont	2010-11	12,912	16.20%	1.07 [0.88, 1.30]	1.26* [1.04, 1.52]	1.69** [1.38, 2.06]	5,031 [b]	39.50%	1.09 [0.91, 1.32]	1.59** [1.30,	2.05** [1.58, 2.65]	12,921	21.80%	1.53** [1.28, 1.82]	2.71** [2.29, 3.22]	4.81** [3.98, 5.80]
Iowa	2012	6,214	12.10%	1.21 [0.89, 1.64]	1.38* [1.03, 1.85]	2.26** [1.64, 3.11]	4,564	39.50%	1.33** [1.11,	1.24* [1.01, 1.52]	1.55** [1.20, 2.00]	6,216	17.10%	2.02** [1.58, 2.60]	2.84** [2.23, 3.61]	5.28** [4.09, 6.81]
Wisconsin	2010-12	13,123	12.10%	1.29* [1.03, 1.62]	1.38** [1.10, 1.74]	1.94** [1.50, 2.52]	9,740	40.30%	1.42** [1.22,	1.48** [1.25,	2.23** [1.78, 2.79]	13,121	16.10%	1.58** [1.29, 1.94]	2.71** [2.24, 3.29]	4.54** [3.64, 5.65]
Minnesota	2011	13,472	10.90%	1.42** [1.12, 1.80]	1.61** [1.27, 2.04]	2.22** [1.71, 2.86]	9,215	32.60%	1.14 [0.97, 1.33]	1.38** [1.17,	1.51** [1.20, 1.91]	13,454	15.40%	1.55** [1.26, 1.92]	2.82** [2.30, 3.45]	5.07** [4.07, 6.31]
Montana	2011	9,149	13.30%	1.11 [0.85, 1.46]	1.50** [1.18, 1.92]	2.10** [1.59, 2.77]	6,843	39.90%	1.17 [0.96, 1.43]	1.38** [1.12,	2.25** [1.73, 2.91]	9,144	21.00%	1.73** [1.35, 2.21]	2.82** [2.23, 3.55]	5.01** [3.93, 6.39]
Nevada	2010	3,471	14.50%	1.17 [0.73, 1.87]	1.96** [1.25, 3.06]	2.18** [1.36, 3.50]	-					3,489	15.70%	1.71* [1.00, 2.91]	4.21** [2.64, 6.72]	6.80** [4.22,
Hawaii	2010	6,081	17.40%	1.26 [0.96, 1.67]	1.22 [0.91, 1.62]	1.70** [1.23, 2.34]	-					6,118	8.90%	1.93** [1.31, 2.83]	3.74** [2.60, 5.36]	6.30** [4.35, 9.13]
Washington	2011	13,575	14.60%	1.05 [0.83, 1.32]	1.38** [1.11, 1.73]	2.00** [1.58, 2.53]	10,560	37%	1.26** [1.08,	1.49** [1.27,	1.56** [1.28, 1.89]	13,571	19.70%	1.67** [1.34, 2.09]	2.71** [2.21, 3.33]	4.62** [3.71, 5.75]
		COPD					Any Cardiovascular Disease					Cancer				
Arkansas	2009	-					2,829	17.50%	1.04 [0.76, 1.43]	0.82 [0.58, 1.17]	1.38 [0.87, 2.18]	2,824	17.20%	0.84 [0.63, 1.14]	1.16 [0.83, 1.62]	0.85 [0.54, 1.34]
Louisiana	2009	-					6,168	17.20%	1.23* [1.00, 1.51]	1.10 [0.87, 1.39]	1.24 [0.89, 1.71]	6,161	14.40%	1.20 [0.97, 1.48]	0.94 [0.73, 1.21]	1.30 [0.93, 1.80]
N.Carolina	2012	-					7,520	14.70%	1.22 [0.98, 1.50]	1.27 [1.00, 1.60]	1.49** [1.11, 1.98]	7,520	20.10%	1.12 [0.93, 1.34]	1.07 [0.86, 1.32]	1.31 [1.00, 1.71]
Tennessee	2012	-					4,227	20.30%	1.18 [0.89, 1.58]	1.13 [0.84, 1.51]	1.24 [0.87, 1.78]	4,227	20.40%	0.98 [0.76, 1.27]	1.02 [0.77, 1.35]	1.18 [0.83, 1.68]
Washington DC	2010	-					2,556	10.30%	0.72 [0.45, 1.14]	1.13 [0.68, 1.86]	1.87* [1.02, 3.41]					
Vermont	2010-11	5057 [b]	7.30%	1.14 [0.79, 1.64]	1.75** [1.20,	3.11** [2.08, 4.65]	10,081	11.60%	0.94 [0.78, 1.14]	1.05 [0.86, 1.28]	1.48** [1.13, 1.95]	5,061 [b]	18.40%	1.20 [0.97, 1.48]	1.11 [0.88, 1.41]	1.50* [1.09, 2.06]
Iowa	2012	-					4,587	14.70%	1.30* [1.02, 1.67]	1.58** [1.20,	2.27** [1.60, 3.22]	4,588	18.30%	1.03 [0.83, 1.27]	1.05 [0.83, 1.33]	1.06 [0.77, 1.47]
Wisconsin	2010-12	3,226 [c]	7.90%	1.44 [0.86, 2.42]	1.48 [0.87, 2.52]	1.73 [0.91, 3.31]	9,786	12.40%	0.97 [0.77, 1.22]	1.14 [0.89, 1.46]	1.41* [1.00, 1.97]	9,783	16.50%	1.26* [1.04, 1.53]	1.68** [1.35, 2.09]	1.47** [1.10, 1.96]
Minnesota	2011	9,229	5.50%	1.31 [0.91, 1.91]	1.53* [1.09, 2.16]	2.34** [1.56,	9,266	11.20%	1.35* [1.06, 1.73]	1.23 [0.95, 1.58]	1.67** [1.17, 2.38]	9,269	17.20%	0.93 [0.76, 1.13]	0.92 [0.75, 1.14]	1.18 [0.90, 1.53]
Montana	2011	6,846	8.50%	1.13 [0.79, 1.61]	1.78** [1.26, 2.52]	2.38** [1.64, 3.46]	6,895	14.10%	1.24 [0.93, 1.65]	0.94 [0.70, 1.27]	1.76** [1.25, 2.48]	6,896	21.30%	0.97 [0.78, 1.21]	1.14 [0.91, 1.43]	1.19 [0.87, 1.61]
Nevada	2010	-					2,616	15.60%	1.19 [0.79, 1.80]	1.37 [0.88, 2.11]	1.49 [0.91, 2.43]					
Hawaii	2010	-					4,703	10.20%	0.94 [0.65, 1.35]	1.12 [0.78, 1.60]	1.91** [1.25, 2.92]					
Washington	2011	10,596	6.20%	1.21 [0.82, 1.79]	1.45* [1.04, 2.02]	1.94** [1.34, 2.79]	10,659	11.9	1.10 [0.86, 1.42]	1.35* [1.05, 1.72]	1.49** [1.12, 2.00]	10,663	18.90%	0.96 [0.80, 1.14]	1.04 [0.87, 1.24]	1.46** [1.17, 1.83]
		Diabetes														
Arkansas	2009	3,595	10.30%	1.08 [0.78, 1.50]	1.02 [0.72, 1.44]	1.63 [1.00, 2.66]										
Louisiana	2009	8,214	11.00%	1.14 [0.90, 1.44]	1.01 [0.78, 1.30]	1.08 [0.76, 1.52]										
N.Carolina	2012	10,745	10.90%	0.85 [0.70, 1.03]	1.28* [1.04, 1.58]	1.24 [0.95, 1.63]										
Tennessee	2012	5,638	12.30%	1.14 [0.87, 1.50]	1.36* [1.03, 1.80]	1.13 [0.80, 1.61]										
Washington DC	2010	3,527	8.10%	0.93 [0.61, 1.40]	0.55** [0.36, 0.84]	1.24 [0.73, 2.08]										
Vermont	2010-11	12,964	7.20%	0.90 [0.74, 1.10]	1.04 [0.85, 1.29]	1.34* [1.04, 1.73]										
Iowa	2012	6,226	9.70%	1.18 [0.93, 1.50]	1.01 [0.77, 1.31]	1.75** [1.28, 2.41]										
Wisconsin	2010-12	13,150	7.90%	1.15 [0.93, 1.43]	1.26* [1.01, 1.58]	1.17 [0.78, 1.46]										
Minnesota	2011	13,498	7.30%	1.04 [0.83, 1.30]	1.37* [1.06, 1.77]	1.55** [1.13, 2.12]										
Montana	2011	9,168	8%	1.01 [0.75, 1.36]	1.43* [1.08, 1.90]	1.68** [1.19, 2.37]										
Nevada	2010	3,493	8.50%	1.00 [0.59, 1.71]	0.99 [0.60, 1.63]	0.82 [0.46, 1.46]										
Hawaii	2010	6,128	8.20%	1.11 [0.82, 1.51]	0.78 [0.56, 1.09]	0.64 [0.40, 1.03]										
Washington	2011	13,618	9.40%	1.00 [0.79, 1.28]	1.08 [0.87, 1.35]	1.11 [0.85, 1.46]										

$*p \leq .05$; $**p \leq .01$

Models for chronic disease and depression include baseline controls (see Table 3) and indicators for lifetime smoking, past 30-day heavy drinking, and obesity with flags for missing information

a Number of cases in each state with information on health outcome; sample size for estimation may differ

b Vermont arthritis, COPD, and cancer data from only 2011

c Wisconsin COPD data from only 2011
